# Integrating Screening and Treatment of Unhealthy Alcohol Use and Depression with Screening and Treatment of Anxiety, Pain, and Other Substance Use Among People with HIV and Other High-Risk Persons

**DOI:** 10.1007/s10461-021-03245-5

**Published:** 2021-04-07

**Authors:** Ellen C. Caniglia, Maria Khan, Kaoon Ban, R. Scott Braithwaite

**Affiliations:** Department of Population Health, NYU School of Medicine, 227 E. 30th St., New York, NY 10016 USA

**Keywords:** Alcohol use, Depression, Tobacco, Screening, Treatment

## Abstract

We review and synthesize results from a series of analyses estimating the benefit of screening for unhealthy alcohol use, depression, and tobacco to detect individuals at heightened risk for co-occurring anxiety, pain, depression, unhealthy alcohol use, and other substance use among people with HIV and HIV-uninfected individuals in the Veterans Aging Cohort Study. We also examine the potential impact of *reducing* unhealthy alcohol use and depressive symptoms on the incidence of co-occurring conditions. We found that screening for alcohol and depression may help identify co-occurring symptoms of anxiety, depression, and pain interference, treating unhealthy alcohol use may improve co-occurring pain interference and substance use, and improving depressive symptoms may improve co-occurring anxiety, pain interference, and smoking. We propose that an integrated approach to screening and treatment for unhealthy alcohol use, depression, anxiety, pain, and other substance use may facilitate diagnostic assessment and treatment of these conditions, improving morbidity and mortality.

## Introduction

The health impact of unhealthy alcohol use is substantial and increasing. Alcohol use disorder (AUD) is the most common substance use disorder globally, estimated to affect 100 million individuals, and has increased by 50% in the United States (US) over the past 10 years [1, 2]. Use of substances other than alcohol are also highly prevalent in the US and globally [3]. Tobacco use is the leading cause of preventable death globally, accounting for more than 7 million deaths per year worldwide [4]. The US is in the midst of an unprecedented opioid epidemic [5] and also has experienced recent emergent drug crises [6–9].

Substance use commonly co-occurs with depression [10–12], anxiety [13, 14] and pain [15–20]. Notably, depression is the most common psychiatric disorder globally and the most common mental health condition diagnosed in US primary care settings, impacting physical health and disease prognosis across health conditions [21–25]. Anxiety and pain also affect an increasing number of individuals in the US [26–28]. An established body of research shows that alcohol, depression, anxiety, pain, and other substance use often cluster together, and an emergent body of literature is showing how they are inextricably linked [1, 26–29]. Individuals with one condition are likely to have a second condition, and resolution of one condition may increase the likelihood of a second condition resolving [30, 31]. Interventions to reduce these conditions may substantially reduce the burden of preventable morbidity and mortality in the United States [32].

Substance use, psychiatric, and pain conditions are often more prevalent in people living with HIV than in HIV-uninfected individuals, and lead to poorer HIV outcomes [33–37]. Generalized anxiety disorder (GAD) occurs in 19% of people living with HIV, compared with 2.7% in the general US population, and is associated with poorer engagement in the HIV care continuum (use of antiretroviral therapy, adherence, and viral suppression) [38]. Chronic pain may impact as many as 54% of individuals living with HIV [37], and people with HIV may be twice as likely to be diagnosed with depression compared with people without HIV [39]. Depression, chronic pain, and substance use are also linked to barriers to viral suppression and optimal clinical outcomes [36, 40–44]. Similarly, co-occurring depression, chronic pain, and substance use, may be more likely among individuals with HCV as compared with individuals without HCV [45–50].

The U.S. Preventive Services Task Force recommends screening for alcohol use, depression, and tobacco use in clinical practice in the United States [51], and recent estimates suggest uptake of these screening guidelines is moderate to high [52–55]. Anxiety, pain, and other substance use are not routinely assessed in clinical practice though new guidelines recommend routine screening for illicit drug use among those 18 years or older, when linkage to treatment and care can be offered [56]. Screening for these and other interlinked conditions in routine clinical practice may not be practical due to time, cost, and other constraints. However, given the strong correlations between these clustering conditions, screening for alcohol, depression, and tobacco could identify individuals at high-risk for a clustering condition, at no extra time, cost or response burden. Specifically, routine screening algorithms could be leveraged to facilitate diagnostic assessment and treatment of clustering conditions that may otherwise be undetected and untreated.

In a series of published and unpublished analyses, we estimated the benefit of screening for unhealthy alcohol use [57], depression [58], and tobacco to detect individuals at heightened risk for co-occurring anxiety, pain, depression, unhealthy alcohol use, and other substance use among people with HIV and HIV-uninfected individuals in the Veterans Aging Cohort Study (VACS), a longitudinal study of veterans receiving care in the Veterans Affairs (VA) healthcare system. We evaluated common screening tools used in clinical practice: the Alcohol Use Disorders Identification Test (AUDIT) for alcohol screening [59], the Patient Health Questionnaire (PHQ-9) for depression screening [60], and a one-item smoking questionnaire for tobacco screening. Following these analyses, we examined the potential impact of *reducing* unhealthy alcohol use and depressive symptoms on the incidence of co-occurring conditions in two follow-up studies [31]. Here, we briefly review the methodology used in this body of work, synthesize our main results, apply our results to key subgroups defined by HIV and HCV, and provide suggestions for steps forward.

## Methods

The study sample, measures, and analytic approach have been described in detail previously [57, 58]. Briefly, the VACS survey sample includes US veterans receiving healthcare in 8 VA centers: Atlanta, Baltimore, Dallas, Houston, Los Angeles, New York (Manhattan, Brooklyn, and the Bronx), Pittsburgh, and Washington, D.C. The VACS sample is comprised of approximately equal numbers of patients with HIV and without HIV in primary care practices who are matched by age, race, gender, and VA center of care. We used data from up to 8 annual surveys (depending on when each screening tool was administered), administered between 2003 and 2015.

Alcohol use was measured using the Alcohol Use Disorder Identification Test (AUDIT) and a score of 8 or more was considered as indicative of unhealthy alcohol use. Depressive symptoms were measured using the PHQ-9 instrument and a score of 10 or more was considered indicative of current depressive symptoms. Pain interference symptoms were assessed using one question from the Health Survey Short-Form 12 that asks: “During the last month, how much has pain interfered with your normal work (including work outside and inside the home)” and responses of “moderately,” “quite a bit,” or “extremely” were considered indicative of pain interference symptoms. Anxiety symptoms were assessed by a single survey item asking if the participant had “felt nervous or anxious” in the 4 weeks before the survey, and responses of “yes” were considered indicative of anxiety symptoms. Any tobacco smoking history and past-year use of marijuana, cocaine, other stimulants, injection drug use, and illicit opioids were also examined using single survey items (See “Appendix” Table for a description of when each screening tool was administered).

In a series of published and unpublished analyses, we assessed the test performance of the AUDIT, the PHQ-9, and the one-item tobacco survey item as screening tools for identifying clustering condition symptoms. We evaluated these tools by calculating the sensitivity, specificity, and likelihood ratio (sensitivity/1-specificity) of each measure to identify pain interference symptoms, depressive symptoms, anxiety symptoms, unhealthy alcohol use, and other substance use. For example, to calculate the sensitivity of the AUDIT as a screening tool to identify anxiety symptoms, we calculated the proportion of individuals who answered yes to the anxiety survey question who also had an AUDIT score of eight or greater.

Here, we apply our previous results [57, 58] to key populations within the VACS cohort. Specifically, we used the first follow-up survey to calculate the baseline prevalence of unhealthy alcohol use, anxiety symptoms, depressive symptoms, pain interference symptoms, cocaine use, and injection drug use in the entire VACS cohort, in people with HIV only, and in people with HCV only. We then apply the likelihood ratios calculated previously to these values to calculate post-test probabilities of anxiety symptoms, depressive symptoms, pain interference symptoms, and substance use given a ‘positive’ screening result using routine screening algorithms (eg., AUDIT, PHQ-9, and the one-item tobacco smoking question).

Finally, after assessing the test performance of the AUDIT and the PHQ-9 to detect clustering condition symptoms, we evaluated the potential impact of *reducing* unhealthy alcohol use and depressive symptoms on reducing the incidence of the same clustering conditions in two follow-up analyses. First, we compared individuals with AUDIT scores of eight or greater who reduced their drinking (AUDIT < 8) with those who did not reduce their drinking after 1-year of follow-up. [31] To do so, we fit separate logistic regression models to estimate odds ratios for improvement of each clustering condition after 2-years of follow-up among individuals who had that condition at baseline: moderate or severe pain interference symptoms, tobacco smoking, cannabis use, cocaine use, depressive symptoms, and anxiety symptoms. Inverse probability weighting was used to account for potential selection bias and confounding by baseline HIV status, race, education, income, AUDIT score, PHQ-9 score, pain interference symptoms, smoking, anxiety symptoms, and cannabis, cocaine, other stimulant, and illicit opioid use. Second, we compared individuals with PHQ-9 scores of 10 or greater whose depressive symptoms were reduced (PHQ-9 < 10) with those whose depressive symptoms were not reduced after 1-year of follow-up. Again, we fit separate logistic regression models to estimate odds ratios for improvement of each clustering condition after 2-years of follow-up among individuals who had that condition at baseline. Here, we present results overall, by HIV status, and by HCV status.

### Compliance with Ethical Standards

The authors report no conflicts of interest. Institution review boards at each VACS study site as well as New York University and Yale University approved all study activities. All study participants provided informed consent at study enrollment.

## Key Results and Interpretation

In our previous analyses, we found that AUDIT scores of ≥ 20 yielded strong test performance as indicated by high likelihood ratio values (the increase in the odds of a condition existing given a positive test result, on the multiplicative scale) for symptoms of depression (3.63), for symptoms of anxiety (3.90) and for cocaine use (6.27) [57]. These likelihood ratio values were comparable to dedicated screening tools for these conditions, perhaps indicating that an AUDIT score ≥ 20 could be used to trigger diagnostic assessment for depression, anxiety and cocaine use. Elevated PHQ-9 scores (using thresholds of ≥ 10 and of ≥ 20) yielded large likelihood ratios for symptoms of pain (4.36 and 7.84) and yielded likelihood ratios for symptoms of anxiety (8.24 and 21.57) [58] that potentially outperform the GAD-7, a dedicated screening tool for anxiety. This finding points to a potential opportunity to use the PHQ-9 screener to identify and treat otherwise undiagnosed anxiety and pain. While test specificity was high for AUDIT score ≥ 20 (> 98% for symptoms of depression and anxiety and cocaine use) and for PHQ-9 score ≥ 20 (> 99% for symptoms of anxiety and pain), test sensitivity was quite low, indicating that false negatives using these screening tools would be quite high.

In populations with elevated prevalences of clustering conditions (eg, people with HIV, people with HCV, or non-adherent individuals with chronic conditions), these likelihood ratio values will confer high post-test likelihoods of an individual having the condition of interest. In the VACS sample, the baseline prevalence of anxiety symptoms is 42.20%. Therefore, the estimated likelihood ratios indicate that an individual with an AUDIT score ≥ 20 is estimated to have a 74.01% probability of anxiety symptoms and an individual with a PHQ-9 score ≥ 20 is estimated to have a 94.03% probability of anxiety symptoms (Table 1). In this population, the baseline prevalence of depressive symptoms is 23.20%, indicating that an individual with an AUDIT score ≥ 20 is estimated to have a 52.30% probability of depressive symptoms. The baseline prevalence of pain interference symptoms is 38.70%, which indicates that an individual with a PHQ-9 score ≥ 20 is estimated to have an 83.19% probability of pain interference symptoms. Finally, the baseline prevalence of cocaine use is 10.10%, which indicates that an individual with an AUDIT score ≥ 20 is estimated to have a 41.33% probability of cocaine use. Post-test probabilities are equally large among people with HIV and slightly larger among people with HCV.

**Table 1:** Selected post-test probabilities of anxiety symptoms, depressive symptoms, and pain interference symptoms using screening instruments identified as effective in prior work (AUDIT, PHQ-9 and the one-item tobacco smoking question), overall and in key subgroups

	Baseline prevalence (%)	Post-test probability using AUDIT score ≥ 20 as screening tool (%)	Post-test probability using PHQ-9 score ≥ 20 as screening tool (%)	Post-test probability using no tobacco smoking history^a^ as screening tool (%)
Clustering condition and key population				
Unhealthy alcohol use				
VACS (all)	11.80	–	21.28	4.72
People with HIV only	11.10	–	20.14	4.42
People with HCV only	13.40	–	23.81	5.42
Anxiety symptoms				
VACS (all)	42.20	74.01	94.03	35.08
People with HIV only	44.60	75.84	94.55	37.33
People with HCV only	47.10	77.64	95.05	39.72
Depression symptoms				
VACS (all)	23.20	52.30	–	18.87
People with HIV only	23.20	52.30	–	18.87
People with HCV only	29.70	60.53	–	24.55
Pain interference symptoms				
VACS (all)	38.70	55.18	83.10	32.71
People with HIV only	33.10	49.10	79.50	27.59
People with HCV only	40.50	57.03	84.22	34.39
Cocaine use				
VACS (all)	10.10	41.33	15.24	3.78
People with HIV only	13.30	49.03	19.71	5.10
People with HCV only	19.20	59.84	27.55	7.68
Injection drug use				
VACS (all)	2.00	6.83	3.03	0.53
People with HIV only	3.00	9.99	4.52	0.80
People with HCV only	5.80	18.10	8.61	1.58

^a^Negative likelihood ratios are used to calculate the post-test probability of each condition given no tobacco smoking history

Some findings suggested that failure to endorse a condition can provide clinically meaningful information on who does not need to be screened for a second condition. Specifically, when using the one-item tobacco smoking questionnaire typically used in clinical practice, we observed those who have never smoked versus those endorsing any smoking history had a substantially lower likelihood of other substance use as indicated by low negative likelihood ratios (1 − sensitivity/specificity) for unhealthy alcohol use (0.37), injection drug use (0.26) and crack/cocaine use (0.35). These results suggested it may be justified to reduce intensity of screening for these substances among never-smokers when care settings are resource constrained. Doing so may permit resources for substance use detection and treatment to be more effectively deployed. For example, given the current opioid epidemic, this information could be useful to best allocate resources for opioid misuse detection.

We found some evidence for greater improvement of pain interference symptoms and greater discontinuation of co-occurring substance use (i.e. smoking, cocaine, and cannabis) after reducing AUDIT score to < 8 (“reducing drinking”) compared to retaining an AUDIT score ≥ 8 (not “reducing drinking”) [31]. Among individuals with moderate or severe pain interference symptoms at baseline, the adjusted odds ratio (95% confidence interval) for improvement of moderate or severe pain interference symptoms was 1.49 (0.91, 2.42) after 2-years comparing “reducing drinking” (AUDIT < 8) versus not “reducing drinking” (AUDIT ≥ 8). These odds ratios (95% CIs) were 1.57 (0.93, 2.63) for stopping smoking, 1.65 (0.92, 2.95) for no longer using cannabis, and 1.83 (1.03, 3.27) for no longer using cocaine (Table 2). In subgroup analyses, adjusted odds ratios for discontinuing smoking and cocaine use were larger among people with HIV compared to people without HIV, but were smaller for improvement of pain interference symptoms among people with HIV compared to people without HIV. Results did not vary substantially by HCV status.

**Table 2:** Selected odds ratios and 95% confidence intervals for each condition improving or resolving at 2-years comparing: those who “reduce drinking” (AUDIT < 8) at 1-year with those who do not “reduce drinking” (AUDIT ≥ 8) at 1-year (column 3); and those who reduce depressive symptoms (PHQ-9 < 10) at 1-year with those who do not reduce depressive symptoms (PHQ-9 ≥ 10) at 1-year (column 4), overall and in key subgroups

Clustering condition improves	Population	Odds ratio^a^ (95% CI) for “reducing drinking” versus not “reducing drinking”	Odds ratio^a^ (95% CI) for reducing depressive symptoms versus not reducing depressive symptoms
Moderate or severe pain interference symptoms	VACS (all)	1.49 (0.91, 2.42)	2.15 (1.59, 2.92)
	HIV+	0.93 (0.43, 2.03)	2.28 (1.18, 4.38)
	HIV−	2.03 (1.07, 3.85)	1.71 (1.11, 2.64)
	HCV+	1.36 (0.61, 3.02)	2.15 (1.59, 2.92)
	HCV−	1.80 (0.94, 3.45)	2.51 (1.69, 3.73)
Smoking	VACS (all)	1.57 (0.93, 2.63)	1.46 (0.93, 2.30)
	HIV+	2.44 (1.18, 5.03)	1.95 (0.83, 4.57)
	HIV−	0.86 (0.40, 1.85)	1.69 (0.86, 3.34)
	HCV+	1.77 (0.83, 3.77)	1.46 (0.93, 2.30)
	HCV−	1.37 (0.67, 2.80)	1.47 (0.79, 2.75)
Anxiety symptoms	VACS (all)	1.33 (0.80, 2.22)	3.25 (2.24, 4.73)
	HIV+	1.38 (0.68, 2.80)	2.18 (1.06, 4.51)
	HIV−	1.21 (0.57, 2.56)	4.08 (2.33, 7.13)
	HCV+	1.26 (0.59, 2.68)	3.25 (2.24, 4.73)
	HCV−	1.37 (0.68, 2.78)	4.01 (2.49, 6.44)
Cannabis use	VACS (all)	1.65 (0.92, 2.95)	0.97 (0.60, 1.58)
	HIV+	1.36 (0.65, 2.84)	0.90 (0.33, 2.49)
	HIV−	2.25 (0.86, 5.87)	0.93 (0.46, 1.88)
	HCV+	1.41 (0.58, 3.44)	0.97 (0.60, 1.58)
	HCV−	1.84 (0.85, 4.01)	1.13 (0.60, 2.11)
Cocaine use	VACS (all)	1.83 (1.03, 3.27)	1.27 (0.79, 2.06)
	HIV+	2.12 (1.02, 4.43)	1.39 (0.60, 3.21)
	HIV−	1.42 (0.56, 3.62)	1.28 (0.61, 2.67)
	HCV+	1.79 (0.79, 4.02)	1.27 (0.79, 2.06)
	HCV−	2.15 (0.91, 5.10)	1.28 (0.62, 2.64)

^a^All analyses are restricted to individuals with that co-occurring condition at baseline

Finally, we found some evidence for greater improvement of pain interference and anxiety symptoms and greater discontinuation of smoking after reducing depressive symptoms (PHQ-9 < 10) versus not reducing depressive symptoms (PHQ-9 ≥ 10). Among individuals with moderate or severe pain interference symptoms at baseline, the adjusted odds ratio (95% confidence interval) for improvement of moderate or severe pain interference symptoms was 2.15 (1.59, 2.92) after 2-years comparing reducing depressive symptoms (PHQ-9 < 10) versus not reducing depressive symptoms (PHQ-9 ≥ 10). These odds ratios (95% CIs) were 3.25 (2.24, 4.73) for reducing anxiety symptoms and 1.46 (0.93, 2.30) for stopping smoking (Table 2). In subgroup analyses, the adjusted odds ratio for reducing anxiety symptoms was smaller among people with HIV compared to people without HIV. Results did not vary substantially by HCV status.

## Discussion and Next Steps

The AUDIT and PHQ-9 are commonly used screening tools in clinical practice. Our published and ongoing work provides evidence that screening for alcohol and depression may help identify clustering conditions, especially symptoms of anxiety, depression, and pain interference, and that treating unhealthy alcohol use and depression may in turn improve clustering condition symptoms, especially symptoms of anxiety and pain.

Our work has some limitations. Symptoms of pain interference and anxiety and use of tobacco and other substances were assessed using single-item screening tools. While single-item screeners are not ideal, these measures are widely used in clinical practice [57, 61–63]. In addition, our analyses relied on self-reported substance use variables, which may be influenced by social desirability bias. Finally, our analyses dichotomized unhealthy alcohol use and depression symptoms, though we chose cut-points typically used in clinical care.

It is difficult to identify the optimal cascade of screening and diagnostic tests for unhealthy alcohol use and clustering conditions based on these results alone because the relative benefits, harms, and costs of different strategies need to be iterated, quantified and compared. Accordingly, our group is in progress incorporating these relationships into a decision model that can consider a wide variety of screening and diagnostic strategies, including the consequences of false negative and false positive tests, the response burden of multiple screening questionnaires, and other resource constraints. As an example of insights that may emerge, it is possible that a high AUDIT score should trigger diagnostic assessments for depression, anxiety and for cocaine use if the baseline prevalences of these conditions are sufficiently high. As another example, it may be possible that a high PHQ-9 score should trigger diagnostic assessments for anxiety and pain in certain populations. Because test sensitivity was low for high AUDIT and high PHQ-9 scores, these tools should not be used to replace screening tools for other co-occurring conditions or in isolation.

Integrating screening and treatment for unhealthy alcohol use and depression with screening and treatment of anxiety, pain, and other substance use may facilitate diagnostic assessment and treatment of these conditions, which are often undetected and untreated. The benefits of an integrated approach may be greater among people with HIV (and other populations including people with HCV) with elevated risk of depression, anxiety, and substance use, especially since diagnosing and treating these conditions can lead to improvements in HIV care and in HIV outcomes. We hope that our analyses will illuminate tractable approaches that more effectively mitigate the preventable morbidity and mortality burden from these conditions.

## Appendix

See Table 3.

**Table 3:** Across-time screening tools administered among Veterans Aging Cohort Study Participants (2003–2015)

	Survey waves							
	2003–2004	2004–2005	2005–2007	2008–2009	2009–2011	2011–2012	2012–2014	2015
PHQ-9	x	x	x	x	x	x	x	x
Anxiety symptoms	x	x	x	x		x	x	x
Pain interference symptoms	x	x	x	x	x	x	x	x
AUDIT	x	x	x	x	x	x		
Tobacco smoking	x	x	x	x	x	x	x	x
Marijuana	x	x	x	x	x	x	x	x
Illicit opioids^a^	x	x	x	x	x	x	x	x
Injection drug use	x	x	x	x	x	x	x	x
Cocaine	x	x	x	x	x	x	x	x
Other stimulants^b^	x	x	x	x	x	x	x	x

^a^Includes non-medical use of prescription opioids “such as Oxycontin, Vicodin, Percocet” or heroin use (note: prescription opioids were not assessed during the 2005–2007 survey wave)

^b^Other stimulants defined as “amphetamines, uppers, speed, crank, crystal meth, bam”
